# Effectiveness, safety, and economic evaluation of adjuvant moxibustion therapy for aromatase inhibitor-induced arthralgia of postmenopausal breast cancer stage I to III patients: Study protocol for a prospective, randomized, assessor-blind, usual-care controlled, parallel-group, pilot clinical trial

**DOI:** 10.1097/MD.0000000000017260

**Published:** 2019-09-20

**Authors:** Seungwon Shin, Bo-Hyoung Jang, Seung-Hyeok Park, Jin-Wook Lee, Min Soo Chae, Namhoon Kim, Hae Sun Suh, Sola Han, Sun Young Min, Sun Kyung Baek, Yu Jin Lim, Deok-Sang Hwang

**Affiliations:** a Clinical Trial Center, Korean Medicine Hospital, Kyung Hee University; b Department of Preventive Medicine, College of Korean Medicine, Kyung Hee University; c Department of Clinical Korean Medicine, Graduate School, Kyung Hee University, Seoul; d Pharmaceutical Economics, Outcomes Research and Policy, Pusan National University, Busan; e College of Pharmacy, Pusan National University, Busan; f Department of Surgery, College of Medicine, Kyung Hee University; g Department of Internal Medicine, College of Medicine, Kyung Hee University; h Department of Radiation Oncology, Kyung Hee University Medical Center, Kyung Hee University College of Medicine; i Department of Korean Obstetrics & Gynecology, College of Korean Medicine, Kyung Hee University, Seoul, Republic of Korea.

**Keywords:** aromatase inhibitors, arthralgia, breast neoplasms, moxibustion, randomized controlled trial, traditional Korean medicine

## Abstract

**Introduction:**

This study is a prospective, assessor-blinded, parallel-group, randomized controlled pilot trial to explore the effectiveness of 12-week adjuvant moxibustion therapy for arthralgia in menopausal females at stage I to III breast cancer on aromatase inhibitor (AI) administration, compared with those receiving usual care.

**Methods/design:**

Forty-six menopausal female patients with breast cancer who completed cancer therapy will be randomly allocated to either adjuvant moxibustion or usual care groups with a 1:1 allocation ratio. The intervention group will undergo 24 sessions of adjuvant moxibustion therapy with usual care for 12 weeks, whereas the control group will receive only usual care during the same period. The usual care consists of acetaminophen administration on demand and self-directed exercise education to manage AI-related joint pain. The primary outcome is the mean change of the worst pain level according to the Brief Pain Inventory—Short Form between the initial visit and the endpoint. The mean changes in depression, fatigue, and quality of life will also be compared between groups. Safety and pharmacoeconomic evaluations will also be included.

**Discussion:**

Continuous variables will be compared by an independent *t* test or Wilcoxon rank-sum test between the adjuvant moxibustion and usual care groups. Adverse events will be analyzed using the chi-square or Fisher exact test. The statistical analysis will be performed by a 2-tailed test at a significance level of .05.

## 1 Introduction

Postmenopausal women with hormone receptor-positive breast cancer receive adjuvant aromatase inhibitors (AIs) for 5 years^[1]^ to suppress recurrence of breast tumor tissue.^[2]^ However, patients receiving AIs commonly develop arthralgia related to AIs, which affects their adjuvant cancer therapy, and also their quality of life (QoL).^[3]^

Aromatase inhibitor-induced arthralgia (AIA) is defined as joint pain which initiates or worsens after AI administration. The joint pain improves or resolves within 2 weeks after AI cessation, but comes back on re-administration of AIs.^[3]^ Patients usually complain of symmetric arthralgia, especially in the hands or wrist joints. The incidence of AIA varies between 5% and 35%. AIA is highly suspected to substantially lead to nonadherence of AI therapy, which could significantly affect breast cancer recurrence.^[3,4]^

Physicians are encouraged to inform patients before initiating AI therapy that they may experience joint pain during AI administration. Exercise, weight loss, vitamin D supply, or selective serotonin reuptake inhibitor administration has been suggested for mild arthralgia, whereas prednisolone or nonsteroidal anti-inflammatory drugs are prescribed for moderate to severe arthralgia in clinical practice.^[3]^ Acupuncture therapy for AIA has also been investigated. A systematic review showed acupuncture therapy has potential benefits in relieving pain in AIA patients.^[4]^ However, standard treatments for AIA are not yet defined.

Moxibustion is a noninvasive procedure in traditional Korean medicine (TKM), which burns moxa (dried mugwort) directly or indirectly on acupoints to alleviate symptoms. It has been widely used in clinical practice for various conditions including rheumatic diseases, dyspepsia, hot flush, and fetal breech presentation. Clinical research shows that moxibustion could improve pain or QoL of knee osteoarthritis patients.^[5–7]^ Another randomized controlled trial (RCT) showed that moxibustion improved anorexia in patients with metastatic cancer.^[8]^ These previous studies imply a possible benefit in administering moxibustion for AIA in breast cancer patients.

This pilot study aims to evaluate the effectiveness and safety of adjuvant moxibustion therapy for postmenopausal female patients with breast cancer stage I to III who complain of joint pain associated with 3 months or more of AI administration.

## 2 Methods/design

This trial protocol conforms to Standard Protocol Items: Recommendations for Interventional Trials (SPIRIT) 2013 statement,^[9]^ and Standard Protocol Items for Clinical Trials with Traditional Chinese Medicine 2018: Recommendations, Explanation and Elaboration (SPIRIT-TCM Extension 2018).^[10]^

### 2.1 Objectives

This study aims to primarily to explore the effectiveness of 12-week adjuvant moxibustion therapy for arthralgia in menopausal females with stage I to III breast cancer under AI administration, compared with those receiving usual care. Further, we will assess how moxibustion therapy affects depression, fatigue, and QoL of AIA patients. Furthermore, the safety and economic evaluation of moxibustion therapy for AIA patients will be performed with this pilot trial.

### 2.2 Study design and setting

This study is a prospective, assessor-blinded, parallel-group, randomized, controlled pilot trial. We will enroll 46 menopausal female patients (aged 40–65) diagnosed with stage I to III breast cancer who have completed cancer therapy and are receiving AIs for 3 months or more. The patients presenting with joint pain with 4 points or more on the Brief Pain Inventory—Short Form (BPI-SF) will be eligible to participate in this trial. After screening assessment and group allocation, they will receive 24 sessions of adjuvant moxibustion therapy with usual care for 12 weeks, whereas the control group will receive only usual care during the same period. The usual care consists of acetaminophen administration and self-exercise to manage AI-related joint pain.

Eligible participants will be enrolled from among the outpatients in the Kyung Hee University Korean Medicine Hospital, Seoul, Republic of Korea. The trial will be advertised via web pages and notice boards for potential participants. Study coordinators will keep closely in touch with the participants to promote adherence.

**Figure 1:**
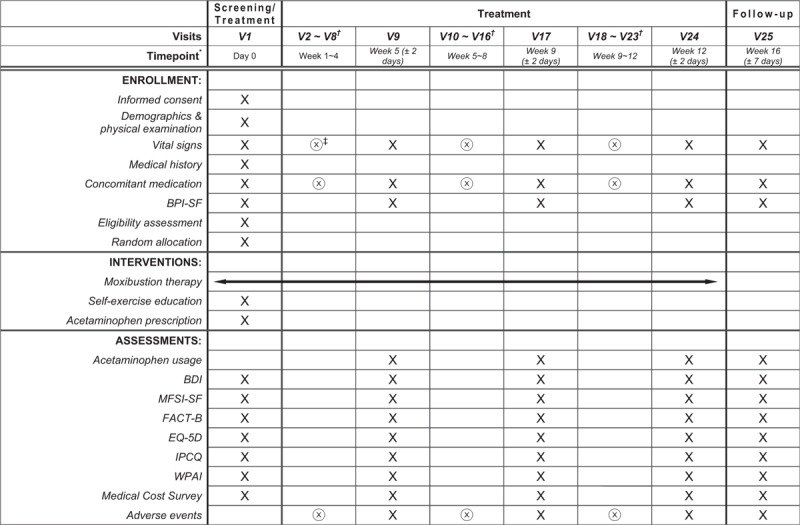
Study flow. ^∗^ Moxibustion therapy will start on the screening day if a participant is found eligible. Patients allocated to the intervention group will visit twice a week for moxibustion therapy (24 sessions for 12 weeks in total). ^†^ Participants should take all corresponding examinations, therapy sessions, and assessments on every visit during V2 ∼ V8, V10 ∼ V16, and V18 ∼ V23. ^‡^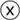 means that the corresponding procedure is only for the participants allocated to the intervention group, while X is for all participants. BDI = Beck Depression Inventory, BPI-SF = Brief Pain Inventory—Short Form, EQ-5D = EuroQoL Five Dimensions Questionnaire, FACT-B = Functional Assessment of Cancer Therapy–Breast, IPCQ = iMTA Productivity Cost Questionnaire, MFSI-SF = Multidimensional Fatigue Symptom Inventory—Short Form, V = Visit, WPAI = Work Productivity and Activity Impairment Questionnaire.

The flow chart following the SPIRIT statement is illustrated in Fig. 1.

### 2.3 Eligibility criteria

The inclusion criteria are as follows:

a) Females with amenorrhea, aged 45 to 65, who are without menstrual period for 1 year or more or with serum follicle-stimulating hormone of 45 mIU/mL or higher;
b) Estrogen receptor-positive breast cancer patients of stage I to III, who have completed surgical procedures, chemotherapy, and/or radiation therapy and do not have active cancer treatment plans;
c) Patients who are taking third-generation AIs such as anastrozole, letrozole, and so on for 3 months or more and are not supposed to cease the administration during the study period;
d) Patients who develop continuing arthralgia in 1 or more joints after AI treatment is initiated;
e) Patients who are assessed with more than 4 points of BPI-SF in terms of the maximum pain level at baseline; and
f) Volunteers who sign the informed consent forms after obtaining all necessary information.

The exclusion criteria are as follows:

a) Patients who have primary or metastatic residual cancer or recurrent cancer;
b) Patients who have had previous fracture or surgery in the painful joints within the past 3 months before enrollment;
c) Patients experiencing moxibustion therapy for AIA within the past 3 months before enrollment;
d) Patients who are receiving steroids or narcotic analgesics at baseline;
e) Patients who elicit an allergic reaction to moxibustion, keloids, or any skin diseases on the targeted acupoints for moxibustion;
f) Patients who are scared of moxibustion therapy; or
g) Patients who have participated in another clinical study within the past month prior enrollment.

### 2.4 Dropout criteria

The participants will be dropped out even after being allocated to a group if they turn out to have one of the following:

a) If investigators find any violation of eligibility criteria after randomization;
b) If participants withdraw consent or refuse moxibustion therapy after randomization;
c) If investigators lose participants to follow-up;
d) If participants cannot continue the study participation due to serious and unexpected adverse events or newly found or worsened concomitant disorders;
e) If participants violate the study protocol including undergoing any prohibited treatments for AIA during study participation; or
f) If participants miss 5 or more sessions of moxibustion therapy.

### 2.5 Randomization, allocation concealment, and blinding

An independent statistician will generate random sequence numbers with the blockrand package (version 1.3) in R (version 3.5.2, The R Foundation). The random number and allocated group will be delivered in sealed envelopes. After completing screening procedures, an investigator will open the sealed envelopes in consecutive order and allocate patients to either the moxibustion group or the usual care group at a 1:1 ratio, enabling complete concealment of group allocation.

This study is an open-label trial; therefore, practitioners and participants cannot be blinded during the whole study. However, an independent outcome assessor will be blinded throughout the study to minimize study bias.

### 2.6 Moxibustion therapy

**Figure 2:**
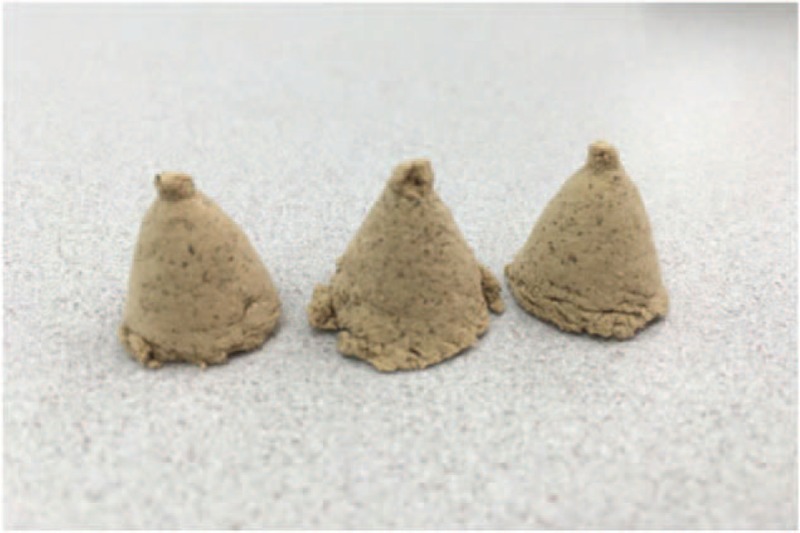
Moxibustion. Dried mugwort in a cone shape has approximately a 2 cm diameter on the base and approximately 1.5 cm in height.

Participants allocated to the intervention group will receive moxibustion therapy twice a week for 12 weeks (24 sessions). Dried mugwort in a cone shape (approximately 2 cm diameter on the base and approximately 1.5 cm height; Dongbang Medical Co. Ltd., Seongnam-si, Gyeonggi-do, Republic of Korea; Fig. 2) will be placed directly on prespecified acupoints of body skin. The dried mugwort will be lit and removed after it burns two-thirds of its height from the top, which will be repeated 3 times on every acupoint.

**Figure 3:**
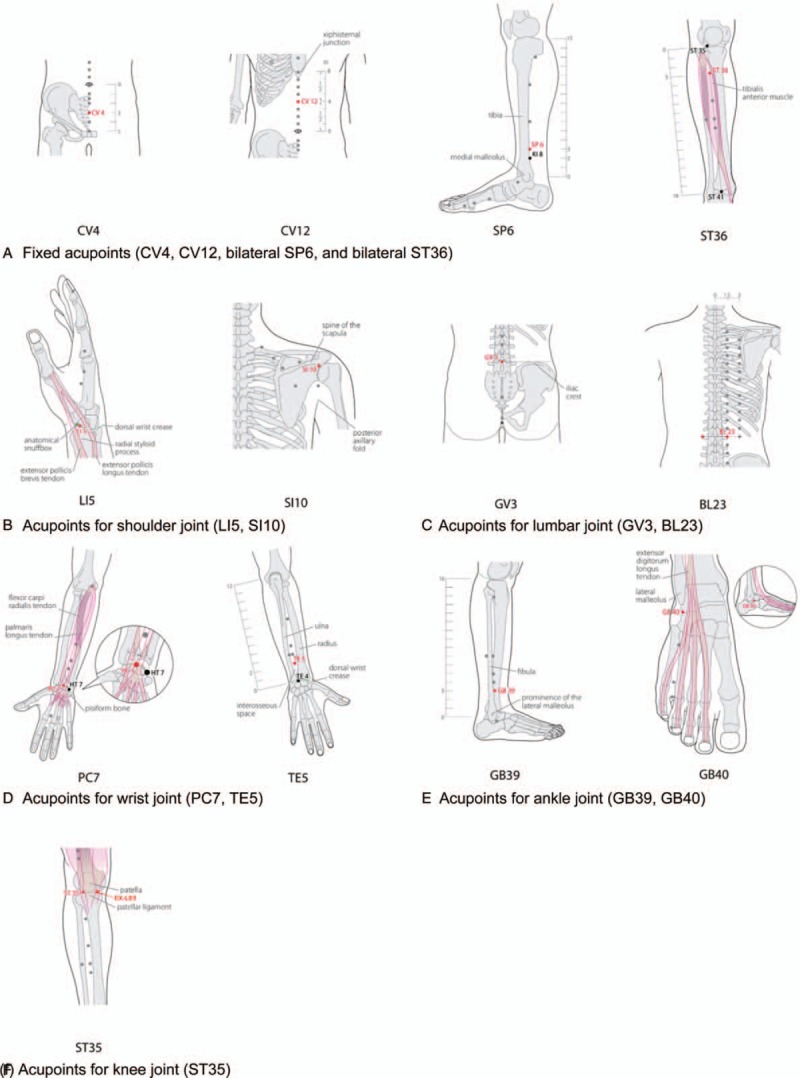
Acupoints for moxibustion therapy. The acupoints for moxibustion therapy consist of fixed acupoints (CV4, CV12, bilateral SP6, and bilateral SP6) and individualized acupoints for corresponding joints with pain. No additional acupoints are selected for cervical joint pain. The locations of each acupoint are extracted from WHO Standard Acupuncture Point Locations in the Western Pacific Region.

The acupoints consist of 6 fixed acupoints (CV4, CV12, bilateral SP6, and bilateral SP6) and individually selected acupoints of the 2 joints with pain except for the cervical joint. If a patient complains of pain in more than 2 joints, only the 2 joints with the most severe pain will be selected for the moxibustion therapy. The prespecified acupoints for each joint are illustrated in Fig. 3. The acupoints have been selected based on previous studies,^[8,11]^ an acupuncture textbook,^[12]^ and specialists’ opinion. The locations of the acupoints will be followed from WHO Standard Acupuncture Point Locations in the Western Pacific Region (Fig. 3).^[13]^ The practitioners will be TKM doctors who have more than 1 year of experience in clinical practice. They will not be allowed patient interaction during therapy sessions to minimize a bias.

### 2.7 Usual care: self-directed exercise education and acetaminophen administration on demand

Participants in the study will all receive usual care for AIA; the intervention group will receive both moxibustion therapy and usual care, and only usual care will be administered to the control group. The control group is set up to represent actual practice.

We will educate the eligible participants with a brochure after their screening assessment, which includes a self-directed exercise program (stretching and walking). Moreover, acetaminophen (500 mg Tylenol tablet, Janssen Korea, Seoul, Republic of Korea) will be prescribed at the initial visit and the patients will be advised to intake the medication appropriately as needed. They may take 1 tablet each time, up to 4 times a day. Because an on-demand policy of acetaminophen administration is planned, we will record how many tablets are consumed based on the number of prescriptions and returns. Furthermore, the patients will be advised to maintain a diary of the drug usage and submit it to the investigator.

All other concomitant treatments for AIA will be strictly prohibited. These include steroid or narcotic analgesics for arthralgia (corticosteroid injection, tramadol, codeine, propoxyphene, oxymorphone, oxycodone, Oxytrex, fentanyl, morphine sulphate, etc), physical therapy (hot pack, infrared therapy, etc), or other TKM treatments (herbal medications, manual or electrical acupuncture, pharmacopuncture, cupping, etc).

### 2.8 Outcomes

Data will be collected and recorded in case report forms. An independent monitor will regularly visit the study site for source data verification, investigator product management, protocol deviation check, and safety issue monitoring.

#### 2.8.1 Primary outcome

The BPI-SF is widely used to evaluate joint pain in breast cancer patients with AIA.^[14]^ The BPI-SF consists of 9 domains and evaluates the severity of pain and its impact on functioning. Patients respond to each question with a numeric rating scale of 1 to 10. A higher score defines more severe pain.^[15]^ The primary outcome is the mean change of the worst pain level in BPI-SF between the baseline (visit 1) and the endpoint (visit 24).

#### 2.8.2 Secondary outcomes

The mean changes in the worst pain level in BPI-SF will be compared between visits 1 and 9, visits 1 and 17, and visit 1 and the follow-up visit (visit 25), respectively. Moreover, the least pain score, the average pain score, the present pain score, the relief level after treatment, and the level of interference to general activity, mood, walking ability, normal work, social relationships, sleep, or social functioning owing to joint pain obtained from BPI-SF assessments will be also compared between groups (visits 1 and 9, visits 1 and 17, visits 1 and 24, and visits 1 and 25, respectively). The amount of acetaminophen administration will be investigated to evaluate the pain severity secondarily. More severe pain will be defined by increased consumption of the medication.

The Beck Depression Inventory (BDI) will be used to evaluate the severity of depression based on 21 items (0–3 points for each item, 63 points in total). We will use the validated Korean version of the BDI for this study.^[16,17]^

The Multidimensional Fatigue Symptom Inventory—Short Form (MFSI-SF) is a 30-item patient-reported outcome tool to evaluate the severity of fatigue. This tool has often been used in cancer patients. The total fatigue score is calculated from 5 subdomains, which is the sum of the general scale score, physical scale score, emotional scale score, and mental scale score minus vigor scale score.^[18]^

Furthermore, we will assess the changes of QoL in breast cancer patients affected by moxibustion therapy with the Functional Assessment of Cancer Therapy–Breast (FACT-B) and EuroQoL Five Dimensions (EQ-5D) questionnaires. FACT-B was developed to evaluate QoL in breast cancer patients and assesses physical, social/family, emotional, and functional well-being based on 36 components. The patients should select a score between 0 and 4 for each component. The Korean version of FACT-B is validated.^[19]^ EQ-5D consists of questions on mobility, self-care, usual activities, pain/discomfort and anxiety/depression, and a visual analog scale to evaluate QoL of the general population. This tool has been also validated in Korean.^[20]^

The mean changes in BDI, MFSI-SF, FACT-B, and EQ-5D between visits 1 and 9, visits 1 and 17, visits 1 and 24, and visits 1 and 25, respectively, will be compared.

#### 2.8.3 Safety assessment

Adverse events will be reported throughout the study period. The frequency of adverse events will be used to assess the safety of adjuvant moxibustion therapy compared with the control group.

#### 2.8.4 Pharmacoeconomic evaluation

The incremental cost-utility ratio will be estimated with the quality-adjusted life-year (QALY), which will be deduced from the EQ-5D and cost data. The incremental cost-effectiveness ratio will also be estimated with BPI-SF scores and cost data. The cost data will be directly obtained from a cost survey of study participants and indirectly from national health insurance resources.

The Work Productivity and Activity Impairment Questionnaire (WPAI) and iMTA Productivity Cost Questionnaire (iPCQ) will assess productivity losses related to health problems, particularly breast cancer in this trial. Patients will respond to 6 items in the WPAI recalling the previous 1 week, and 12 items in the iPCQ recalling the previous 4 weeks.^[21]^

### 2.9 Hypothesis and sample size

The primary outcome is the mean change of the worst pain level in the BPI-SF score after 12 weeks of treatment. The null hypothesis is *μ*_*interventon*_ = *μ*_*control*_, where *μ* denotes the mean change of the worst pain level in BPI-SF scores between the baseline and week 12. Because there was no previous study evaluating the effectiveness of moxibustion therapy for AIA in breast cancer patients, we used an acupuncture study assessing pain severity with BPI-SF for AIA in breast cancer patients. They showed a BPI-SF change of −3.7 ± 2.43 in acupuncture group, and −0.11 ± 3.15 in the sham group between the baseline and the endpoint.^[22]^ The optimal sample size has been calculated as 23 per group, assuming correlation coefficient of 0.1, dropout rate of 0.2, significance level of 0.05, power of 0.95, and a 2-sided test with PASS version 14.0.11 (NCSS Statistical Software). Therefore, 46 eligible participants will be enrolled for the whole study at a 1:1 allocation ratio.

### 2.10 Statistical analysis

The primary analysis will be carried out with the full analysis set (FAS). The FAS is defined as the set of participants who receive 1 or more sessions of moxibustion therapy and assess their BPI-SF at least once. The per protocol set (PPS) will also be analyzed. The PPS is defined as the set of participants who complete all procedures planned in the study.

Continuous variables will be compared by an independent *t* test or the Wilcoxon rank-sum test depending on the normality of the distribution to ascertain statistically significant differences between the intervention and control groups. Adverse events will be compared with the chi-square or Fisher exact test depending on the number of cells which contain values of 5 or less in the contingency table. The statistical analysis will be performed with R (version 3.5.2 or later) at a significance level of 0.05 (2-sided).

### 2.11 Ethics and dissemination

The study protocol and the informed consent form have been reviewed and approved by the Institutional Review Board at Kyung Hee University Korean Medicine Hospital, Republic of Korea (KOMCIRB 2017–08–029) on September 26, 2017 (Protocol V1.1) and registered in the Clinical Research Information Service (CRIS, https://cris.nih.go.kr/cris/en/, KCT0003698) on April 1, 2019. Any amendments of protocol will be published via CRIS after obtaining the approval of the Institutional Review Board at Kyung Hee University Korean Medicine Hospital.

The TKM doctors will explain the necessary information to participants and obtain written informed consent. The personal information of the participants will be protected by random numbers and their initials. Any personal information, including identification code and names, will neither be recorded in case report forms nor shared with others.

The datasets used and/or analyzed after completing the current study will be available from the corresponding author under reasonable requests. The investigators will disseminate the study results and implications via publication.

## 3 Discussion

This study is a prospective, assessor-blinded, parallel-group, randomized controlled pilot trial to explore the effectiveness of a 12-week adjuvant moxibustion therapy in menopausal females with arthralgia at stage I to III breast cancer on AI administration, compared with those receiving usual care. Forty-six menopausal female patients with breast cancer who have completed cancer therapy will be allocated to either an adjuvant moxibustion or usual care group with a 1:1 allocation ratio. The intervention group will receive 24 sessions of adjuvant moxibustion therapy with usual care for 12 weeks, whereas the control group will receive only usual care during the same period. The usual care consists of acetaminophen administration on demand and self-exercise education to manage the AI-related joint pain.

Moxibustion therapy has been widely used in East Asian countries. It is similar to acupuncture therapy in terms of the meridian system and acupoint theories. However, it is different from acupuncture therapy in that moxa is burned on acupoints. Moxibustion therapy is known to bring warmth back to the Yang and eliminate cold in the Yin in traditional theory, resulting in alleviating joint pain.^[23]^ These theories have led to many clinical research studies, particularly for knee osteoarthritis.^[6,7,24]^ We expect this pilot study could find a clinical use for moxibustion therapy in treating AIA in menopausal patients with breast cancer.

## Author contributions

**Conceptualization:** Seungwon Shin, Sun Kyung Baek, Deok-Sang Hwang.

**Data curation:** Seungwon Shin, Deok-Sang Hwang.

**Funding acquisition:** Deok-Sang Hwang.

**Investigation:** Bo-Hyoung Jang, Seung-Hyeok Park, Jin-Wook Lee, Min Soo Chae, Namhoon Kim, Hae Sun Suh, Sola Han, Sun Young Min, Sun Kyung Baek, Yu Jin Lim, Deok-Sang Hwang.

**Methodology:** Seungwon Shin, Bo-Hyoung Jang, Hae Sun Suh, Sola Han, Sun Young Min, Sun Kyung Baek, Deok-Sang Hwang.

**Project administration:** Deok-Sang Hwang.

**Supervision:** Deok-Sang Hwang.

**Writing – original draft:** Seungwon Shin.

**Writing – review & editing:** Seungwon Shin, Seung-Hyeok Park, Jin-Wook Lee, Min Soo Chae, Namhoon Kim, Hae Sun Suh, Sola Han, Sun Young Min, Sun Kyung Baek, Yu Jin Lim, Deok-Sang Hwang.

Deok-Sang Hwang orcid: 0000-0001-9179-0797.
